# A review on robotic fish enabled by ionic polymer–metal composite artificial muscles

**DOI:** 10.1186/s40638-017-0081-3

**Published:** 2017-12-16

**Authors:** Zheng Chen

**Affiliations:** Department of Mechanical Engineering, University of Houston, 4726 Calhoun Road, Room N207, Houston, TX 77204-4006 USA

**Keywords:** Ionic polymer–metal composite, Bio-inspired robotic fish, Dynamic modeling, Fabrication

## Abstract

A novel actuating material, which is lightweight, soft, and capable of generating large flapping motion under electrical stimuli, is highly desirable to build energy-efficient and maneuverable bio-inspired underwater robots. Ionic polymer–metal composites are important category of electroactive polymers, since they can generate large bending motions under low actuation voltages. IPMCs are ideal artificial muscles for small-scale and bio-inspired robots. This paper takes a system perspective to review the recent work on IPMC-enabled underwater robots, from modeling, fabrication, and bio-inspired design perspectives. First, a physics-based and control-oriented model of IPMC actuator will be reviewed. Second, a bio-inspired robotic fish propelled by IPMC caudal fin will be presented and a steady-state speed model of the fish will be demonstrated. Third, a novel fabrication process for 3D actuating membrane will be introduced and a bio-inspired robotic manta ray propelled by two IPMC pectoral fins will be demonstrated. Fourth, a 2D maneuverable robotic fish propelled by multiple IPMC fin will be presented. Last, advantages and challenges of using IPMC artificial muscles in bio-inspired robots will be concluded.

## Introduction

Species invasions, such as Asian carps invasion recently found in the Illinois River, have caused ecological problems for local species [1]. To control the quantity of invasive species, habitat study plays an important role in figuring out an ecological effective way [2]. To enable the study, autonomous, stealthy, and highly maneuverable underwater vehicles are highly desirable in monitoring of the invasive species. Traditional underwater vehicles, such as submarines, are driven by electric motors, which rely on a rotated propeller to generate propulsion. Rotation-based propulsion creates unfavorable acoustic noise, which draws attentions from underwater creatures and thus leads to unfaithful data for their habitat study. More stealthy and environmentally friendly propulsive approaches need to be investigated and adopted for the underwater vehicles in such applications.

After thousand years of evolution, underwater creatures, such as fish and rays, are extremely best swimmers which man-made underwater vehicles cannot compete with. In order to mimic the swimming behavior of biological fish, much effort has been spent on how propulsion is generated by the fish locomotion. For example, Lighthill [3] studied large-amplitude elongated-body theory of fish locomotion. Lauder studied kinematics and dynamics of fish fin [4]. Through those studies, it was found that most of underwater creatures adopt flapping-based propulsion for fast and energy-efficient moving and highly maneuvering through water. Flapping-based propulsion systems have been studied for many years [5–7]. However, in most of cases, the propulsion systems for robotic fish are still driven by electrical motors, which need a power transmission to convert rotation to flapping. Most of power transmission systems are bulky, energy inefficient, and noisy, which are unsuitable for small-size and bio-inspired robots. To avoid using power transmission, a novel actuating material that can naturally generate flapping is greatly needed. It will enable us to design bio-inspired and stealthy robots for ecological underwater monitoring applications.

Electroactive polymers are emerging actuating materials that can generate large deformation under electrical stimuli [8–10]. EAPs win their nickname, artificial muscles, due to their similarities to the biological muscles in terms of achievable stress and strain. EAPs have different configurations and basically they can be divided into two categories: ionic EAPs and dielectric EAPs. Dielectric EAPs are driven by the electrostatic force applied to dielectric polymers, which can generate large contraction [10–12]. Dielectric EAPs require high actuation voltage (typically higher than 1 kV), which limits their applications in underwater bio-inspired robots. Ionic EAPs are driven by the ionic transportation-induced swelling effect, which typically only needs small actuation voltage (1 or 2 V) and can naturally generate bending motion. Ionic polymer–metal composites (IPMCs) are an important category of ionic EAPs due to their chemical stability under wet condition and built-in actuation and sensing capability [9, 13].

**Fig. 1:**
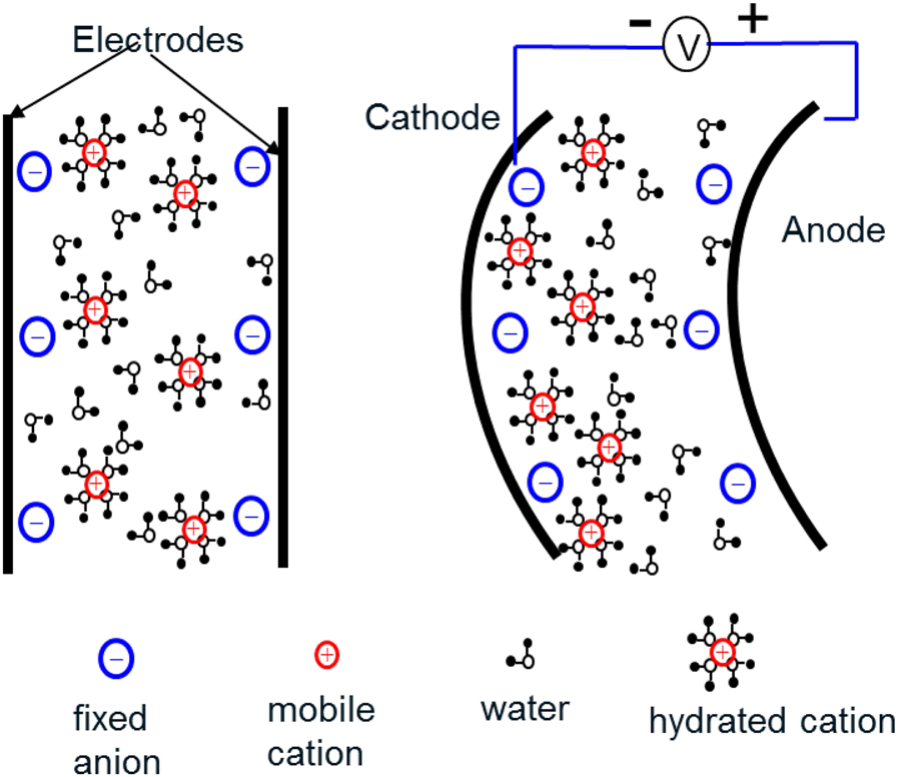
Actuation mechanism of IPMC [14]

An IPMC has a sandwiched structure that consists of an ion exchange membrane coated with two noble metal electrodes, such as gold or platinum, on its surface (Fig. 1) [14]. Application of a small voltage (less than 2 V) to the IPMC creates an electric field that drives the cations (positive ions) to transport to the cathode side while anions (negative ions) are fixed on the carbon polymer chain [15]. The unbalanced cation density distribution along the thickness direction introduces a swelling effect on the cathode side and a shrinking effect on the anode side. Eventually, the IPMC bends to the anode side and thus leads to an actuation effect. Due to their naturally flapping capabilities under wet condition, IPMCs are an ideal engineering actuation material for small-scale and bio-inspired underwater robots.

To achieve a desired actuation performance of IPMC for underwater applications, many researchers have been working on modeling and control of IPMCs. Chen et al. [14] developed a physics-based and control-oriented model for IPMC and then validated the model through designing and implementing a model-based H-infinity control. To accommodate the large system’s uncertainties, such as hydration level, many researchers have developed adaptive robust controls for IPMCs. For example, Anh et al. [16] developed a robust control using quantitative feedback technique (QFT) which identified the system’s characteristics using a pseudorandom binary signal (PRBS) and then a QFT controller was designed and implemented online based on the identified model. Kang et al. [17] developed H-infinity controls with and without loop shaping or μ-synthesis. Their results showed that the robust control techniques can significantly improve the IPMC performance against non-repeatability or parametric uncertainties in terms of the faster response and lower overshoot than the PID control, using lower actuation voltage. Moreover, Chen et al. [18] developed an adaptive control for IPMCs to compensate the hysteresis in IPMC. To avoid using bulky external sensors, many researchers have been focusing on developing a compact sensing scheme for IPMC. For example, Chen et al. developed an IPMC/PVDF sensory actuator and implemented a feedback control using integrated sensing feedback. Leang et al. [19] developed an integrated sensing scheme for IPMCs using strain gauges and then developed a tracking control of an IPMC in an underwater environment.

IPMC-enabled underwater robots have been investigated by many researchers. Guo et al. [20] developed ionic conductive polymer film (ICPF)-enabled robotic fish which can achieve 0.137 body length per second (BL/s) swimming speed. Laurent et al. [21] studied the efficiency of microrobot propelled by IPMC, which can achieve about 1.4% efficiency. Then researchers developed different types of underwater robots, such as robotic fish [22], robotic ray [23], robotic jellyfish [24], and robotic worm [25], for various applications. In this paper, a systems perspective will be taken to review the recent work on IPMC-enabled bio-inspired underwater robots, including (1) a physics-based and control-oriented modeling approach that can capture the intrinsic actuation dynamics of IPMC and the hydrodynamics of robotic fish; (2) a fabrication technology for creating IPMC actuating membranes capable of generating 3D kinematic motions; and (3) bio-inspired design of robotic fish and ray. Finally, discussions and conclusions will be presented at the end.

## Physics-based control-oriented modeling of robotic fish propelled by IPMC caudal fin

Although control of robotic fish powered by electrical motors has been well developed by many research groups [26–32], control of the robotic fish enabled by IPMC has rarely been studied based on our best knowledge. The possible reason might be lacking of a faithful and practical dynamic model of the robotic fish enabled by IPMC. Due to the complex actuation dynamics of IPMC and hydrodynamics of fish, it was a great challenge to get a physics-based control-oriented model. Two types of models have been developed, including a steady-state speed model [33] developed by Tan’s group at Michigan State University and a dynamic model [34] developed by Porfiri’s group at New York University. In this section, we will review the steady-state speed model [33] developed by Tan’s group. In this review, we will discuss Lighthill’s theory on elongated-body propulsion first. Then the IPMC beam dynamics in fluid will be discussed next, considering general force and moment inputs. This will be followed by the actuation model of IPMC caudal fin. Last, the model for computing the speed of IPMC-propelled robotic fish will be obtained by merging Lighthill’s theory and the hybrid tail dynamics. Most of the modeling work presented in this section was published in [14, 33].

### Lighthill theory

**Fig. 2:**
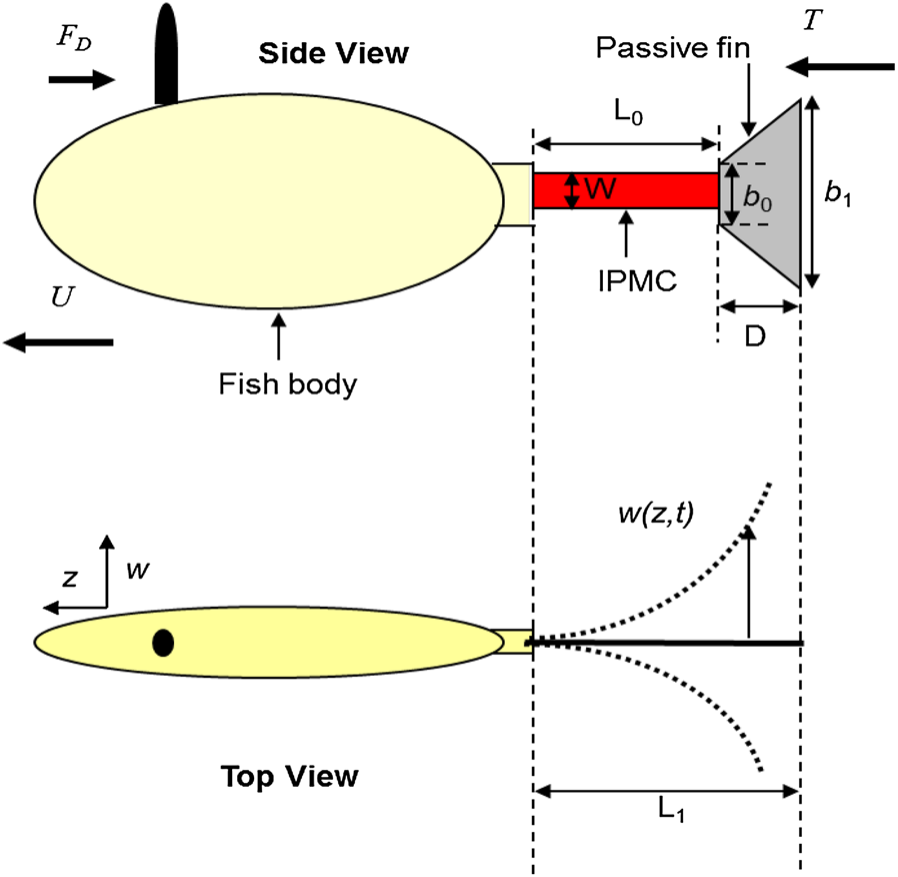
Definition of variables in the robotic fish [33]

A body is considered elongated if its cross-sectional area changes slowly along its length. Suppose that the tail is bending periodically with the bending displacement at z denoted by *w*(*z*, *t*). See Fig. 2 for notation [33]. At the steady state, the fish will achieve a periodic, forward motion with some mean speed *U*. In the discussion here, the word “mean” refers to the average over one period. The mean thrust *T* produced by the tail can be calculated as

1 $$\overline T = \left[{\frac{m}{2} \cdot \left({\overline {\left({\frac{{\partial w\left({z,t} \right)}}{\partial t}} \right)^2} - U^2 \cdot \overline {\left({\frac{{\partial w\left({z,t} \right)}} {\partial z}} \right)^2}} \right)} \right]_{z = L},$$

where *z* = *L*_1_ denotes the end of tail,  denotes the mean value, and *m* is the virtual mass density at *z* = *L*_*1*_, expressed as

2 $$m = \frac{\pi}{4} S_c^2 \rho_w \beta,$$

where *S*_*c*_ is the width of the tail at the end *z* = *L*_*1*_, *ρ*_*w*_ is the fluid density, and *β* is a non-dimensional parameter close to 1. Equation (1) indicates that the mean thrust depends only on the lateral velocity (∂*w*/∂*t*) and the slope (∂*w*/∂*z*) at the tail end. A cruising fish, under inviscid flow conditions, will experience a drag force *F*_D_ as

3 $$F_{\rm D} = \frac{{C_{\rm D} \rho_w U^2 S}}{2},$$

where *S* is the wetted surface area and *C*_D_ is the drag coefficient. At the steady state, the mean thrust *T* is balanced by the drag *F*_D_, from which one can solve the cruising speed *U* as

4 $$U = \left[{\sqrt {\frac{{m \cdot \overline {\left({\frac{\partial w(z,t)}{\partial t}} \right)^2}}}{{C_{\rm D} \rho_w S + m \cdot \overline {\left({\frac{\partial w(z,t)}{\partial z}} \right)^2}}}}} \right]_{z = L}.$$

Since the speed of the fish is related to the lateral velocity and the slope of the trailing edge, one needs to fully understand the actuation dynamics of the tail.

### Model of IPMC hybrid tail

The model combines the seemingly incompatible advantages of both the white-box models (capturing key physics) and the black-box models (amenable to control design). The proposed modeling approach provides an interpretation of the sophisticated physical processes involved in IPMC actuation from a systems perspective. The model development starts from the governing PDE [35, 36] that describes the charge redistribution dynamics under external electrical field, electrostatic interactions, ionic diffusion, and ionic migration along the thickness direction. The model incorporates the effect of distributed surface resistance, which is known to influence the actuation behavior of IPMCs [37]. Moreover, by converting the original PDE into the Laplace domain, an exact solution is obtained, leading to a compact, analytical model in the form of infinite-dimensional transfer function. The model can be further reduced to low-order models, which again carry physical interpretations and are geometrically scalable.

#### Moment generated by IPMC

**Fig. 3:**
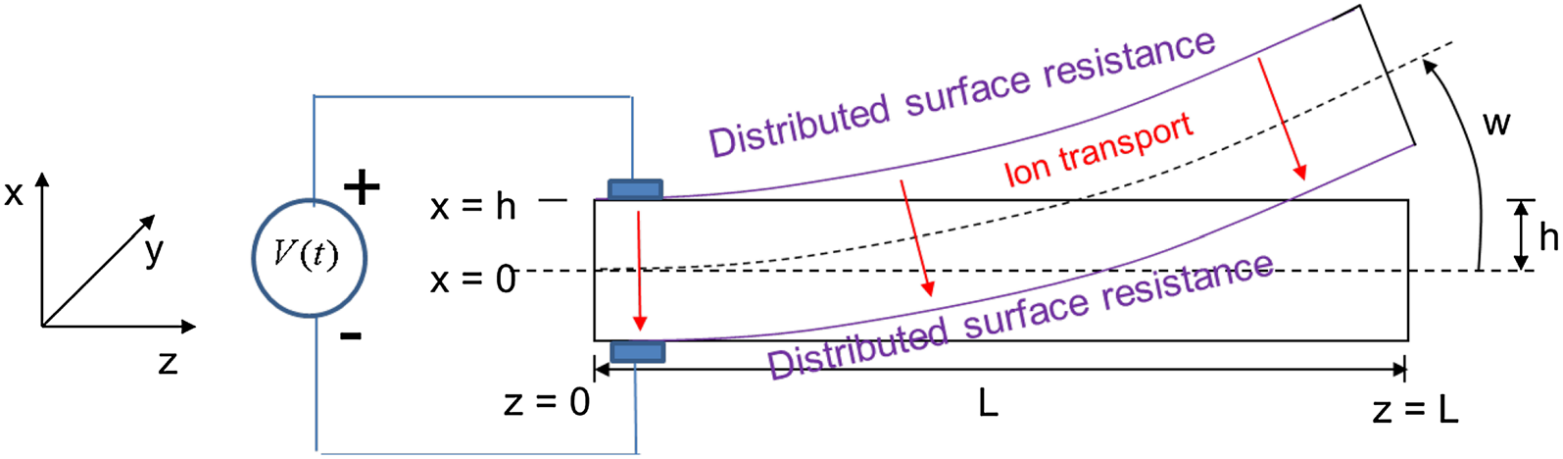
Geometric definitions of an IPMC cantilever beam [14]

Geometric definitions of IPMC cantilever beam are shown in Fig. 3. Let *D*, *E*, *ϕ*, and *ρ* denote the electric displacement, the electric field, the electric potential, and the charge density, respectively. The following equations hold:

5 $$\nabla\cdot \mathbf{D} = \rho = F(C^{+}-C^{-}) ,$$

6 $$\mathbf{E} = -\nabla \phi =\frac{\mathbf{D}}{\kappa_{e}} ,$$

where *κ*_*e*_ is the effective dielectric constant of the polymer, *F* is Faraday’s constant, and *C* ^+^ and *C* ^−^ are the cation and anion concentrations, respectively.The ion transportation can be captured by a second-order linear PDE in terms of charge density [35, 36]:

7 $$\frac{\partial \rho}{\partial t}-d\frac{\partial^2\rho}{\partial x^2} +\frac{F^{2}dC^{-}}{\kappa_{e}RT} \left(1-C^{-}\Delta V\right)\rho =0,$$

Nemat-Nasser and Li assumed that the induced stress is proportional to the charge density [36]

8 $$\sigma = \alpha_0 \rho ,$$

where *α*_*0*_ is the coupling constant. To ease the equation,

9 $$K \stackrel{\bigtriangleup}{=} \frac{F^{2}dC^{-}}{\kappa_{e}RT} \left(1-C^{-}\Delta V\right) .$$

Farinholt investigated the current response of a cantilevered IPMC beam when the base is subject to step and harmonic actuation voltages [36]. A key assumption is that the ion flux at the polymer/metal interface is zero, which serves as a boundary condition for (7), and leads to

10 $$\left. {\left( {\frac{{\partial^{3} \phi }}{{\partial x^{3} }} - \frac{K}{d}\frac{\partial \phi }{\partial x}} \right)} \right|_{x = \pm h} = 0.$$

With distributed surface resistance, we can relate the actuation-induced bending moment *M*_IPMC_(*z*, *s*) at point *z* to the actuation voltage *V*(*s*) by an infinite-dimensional transfer function [14] as

11 $$M_{\rm IPMC} \left( {z,s} \right) = \frac{{\alpha_0 WKk_e \left( {\gamma \left( s \right) - \tanh \left( {\gamma \left( s \right)} \right)} \right)}V(s)}{{\left( {s\gamma \left( s \right) + K\tanh \left( {\gamma \left( s \right)} \right)} \right)}} \cdot \frac{{ \cosh \left( {\sqrt {B\left( s \right)} z} \right) - \sinh \left( {\sqrt {B\left( s \right)} z} \right)\tanh \left( {\sqrt {B\left( s \right)} L} \right) }}{1 + r_2 \theta \left( s \right)},$$

with

$$\theta(s) \mathop = \limits^\Delta \frac{Wk_e s \gamma (s) (s+K)} {h(s \gamma (s) +K\tanh \left( {\gamma \left( s \right)} \right))},$$

$$B\left( s \right) \mathop = \limits^\Delta r_1 \left(\frac{\theta \left( s \right)} {{\left( {1 + r_2 \theta \left( s \right)} \right)}} + \frac{2} {R_p }\right),$$

$$\gamma (s)\mathop = \limits^\Delta \sqrt{\frac{s+K}{d}},$$

$$K \mathop = \limits^\Delta \frac{F^{2}dC^{-}}{\kappa_{e}RT} \left(1-C^{-}\Delta V\right),$$

#### Beam dynamics in fluid

In order to obtain the full actuation model of IPMC, Chen et al. started with a fourth-order PDE for the dynamic deflection function *w*(*z*, *t*) [38] as

12 $$YI\frac{{\partial^4 w\left( {z,t} \right)}}{\partial z^4 } + C \frac{\partial w(z,t)}{\partial t}+ \rho_m A\frac{{\partial^2 w\left( {z,t} \right)}}{\partial t^2 } = f\left( {z,t} \right),$$

where *Y*, *I*, *C*, *ρ*_*m*_, and A denote the effective Young’s modulus, the area moment of inertia, the internal damping ratio, the density, and the cross-sectional area of the IPMC beam, respectively, and *f*(*z*, *t*) is the distributed force density acting on the beam.

The force on the beam consists of two components, the hydrodynamic force *F*_hydro_ from water and the driving force *F*_drive_ due to the actuation of IPMC

13 $$F\left( {z,s} \right) = F_{\rm hydro} \left( {z,s} \right) + F_{\rm drive} \left( {z,s} \right).$$

Assuming that amplitude of flapping is small, the hydrodynamic force acting on the IPMC beam can be expressed as [39]

14 $$F_{\rm hydro} \left( {z,s} \right) = - \rho_w \frac{\pi }{4}W^2 s^2 \varGamma_1 \left( \omega \right)w\left( {z,s} \right), \quad 0\leq z \leq L,$$

where *W* is the width of the IPMC beam, *Γ*_1_(*ω*) is the hydrodynamic function for the IPMC beam subject to an oscillation with radial frequency *ω*, and *ρ*_*w*_ is the density of fluid. The hydrodynamic function for a rectangular beam can be represented as [39].

15 $$\varGamma_1(\omega) = \varOmega(R_e)\left[ {1 + \frac{{4iK_1 \left( { - i\sqrt {i R_e } } \right)}}{{\sqrt {iR_e } K_0 \left( { - i\sqrt {iR_e } } \right)}}} \right],$$

where the Reynolds number *R*_*e*_ of a vibrated beam in water is given by

$$R_e = \frac{\rho_w W^2 \omega}{4\eta}.$$

*K*_0_ and *K*_1_ are modified Bessel functions of the third type, Ω(*R*_*e*_) is the correction function associated with the rectangular beam cross section [39], and *η* is the viscosity of fluid.

#### Hydrodynamic force on passive fin

The hydrodynamic force acting on the passive fin can be written as [27]

16 $$f_{\rm tail} \left( {z,s} \right) = - \frac{\pi }{4}\rho_w s^2 b\left( z \right)^2 \varGamma_2 (\omega) w\left( {z,s} \right),\quad L_0\leq z \leq L_1,$$

where *Γ*_2_(*ω*) is the hydrodynamic function of the passive fin. Note that the hydrodynamic force acting on the active IPMC beam has been incorporated in Eq. (12), and therefore, only the hydrodynamic force on the passive fin needs to be considered here. Since the passive fin used is very light, its inertial mass is negligible compared to the propelled virtual fluid mass and is thus ignored in the analysis here. Considering that the passive fin is rigid compared to IPMC, its width *b*(*z*) and deflection *w*(*z*, *s*) can be expressed as

17 $$b\left(z \right) = \frac{b_1 - b_0}{L_1 - L_0}\left({z - L_0} \right) + b_0,$$

18 $$w\left( {z,s} \right)= w\left( {L_0,s} \right) + \frac{\partial w(L_0,s)}{\partial z}\left( {z - L_0} \right),$$

where *b*_0_, *b*_1_, *L*, *L*_0_, and *L*_1_ are defined in Fig. 2. Then, one can calculate the moment introduced by the passive fin: for *L*_0_ ≤ *z* ≤ *L*_1_.

 If we define

19 $$M_{\rm tail} \left(s \right) = \int\limits_{L_0}^{L_1} {f_{\rm tail}} \left({\tau,s} \right)(\tau-L_0) \hbox{d}\tau,$$

20 $$F_{\rm tail} (s) = \int\limits_{L_0}^{L_1} {f_{\rm tail}} \left({\tau,s} \right) \hbox{d}\tau,$$

then (8.35) can be written as

21 $$M_{\rm fin} \left({z,s} \right) = M_{\rm tail} \left(s \right) + F_{{\rm tai}l} \left(s \right)\left({L_0 - z} \right).$$

#### Mode summation method to solve beam equation

Mode summation method is used to solve the beam dynamics equation. According to the mode analysis method, we can express the solution to (12) as the sum of different modes [40] as

22 $$w\left({z,s} \right) = \sum\limits_{i=1}^{\infty} {\varphi_i \left(z \right)q_i \left(s \right)},$$

where *ϕ*_*i*_(*z*) is the beam shape for the *i*th mode and *q*_*i*_(*s*) is the corresponding generalized coordinate. The mode shape *ϕ*_*i*_(*z*) takes the form

23 $$\varphi_i \left(z \right) = \cosh \left({\lambda_i z} \right) - \cos \left({\lambda_i z} \right) - \beta_i \left({\sinh \left({\lambda_i z} \right) - \sin \left({\lambda_i z} \right)} \right),$$

where *λ*_*i*_ can be obtained by solving

24 $$1 + \cos \left({\lambda_i L} \right)\cosh \left({\lambda_i L} \right) = 0,$$

and

25 $$\beta_i =\frac{{\sinh \left({\lambda_i L} \right) - \sin \left({\lambda_i L} \right)}}{{\cosh \left({\lambda_i L} \right) + \cos \left({\lambda_i L} \right)}}.$$

The generalized coordinate *q*_*i*_(*s*) can be represented as

26 $$q_i \left(s \right) = f_i(s)Q_i(s),$$

where *f*_*i*_(*s*) is the generalized force

27 $$Q_{i}(s) = \frac{1}{s^2 + 2\xi_i \omega_i s + \omega_i^2},$$

and the natural frequency *ω*_*i*_ and the damping ratio *ξ*_*i*_ for the *i*th mode are

28 $$\omega_i = \frac{C_i^2}{L^2}\sqrt {\frac{YI}{\mu_v(\omega_i)}},$$

29 $$\xi_i = \frac{C_v(\omega_i)}{2 \mu_v(\omega_i) \omega_i},$$

and *C*_*i*_ = *λ*_*i*_ *L*. Noting that Γ_1_ (*ω*) is almost a constant value in the frequency region around *ω*_*i*_, one can consider *μ*_*v*_(*ω*_*i*_) as a constant in (18). Therefore, *ω*_*i*_ can be obtained approximately. Then, with *ω*_*i*_, *ξ*_*i*_ can be obtained from (29).

The moment *M*_IPMC_(*z*, *s*) can be replaced by actuation by three components: a distributed force density *F*_d_(*z*, *s*) acting along the length, a concentrated force *F*_c_(*L*, *s*), and a moment *M*(*L*, *s*) acting at the IPMC tip *z* = *L*, where

30 $$F_{\rm c} \left({L,s} \right) = - \frac{\partial M_{\rm IPMC}(z,s)}{\partial z}\left|_{z=L} \right.,$$

31 $$F_{\rm d}(z,s) = \frac{\partial^2 M_{\rm{IPMC}}(z,s)}{\partial z^2},$$

32 $$M(L,s) = M_{\rm IPMC}(L,s).$$

**Fig. 4:**
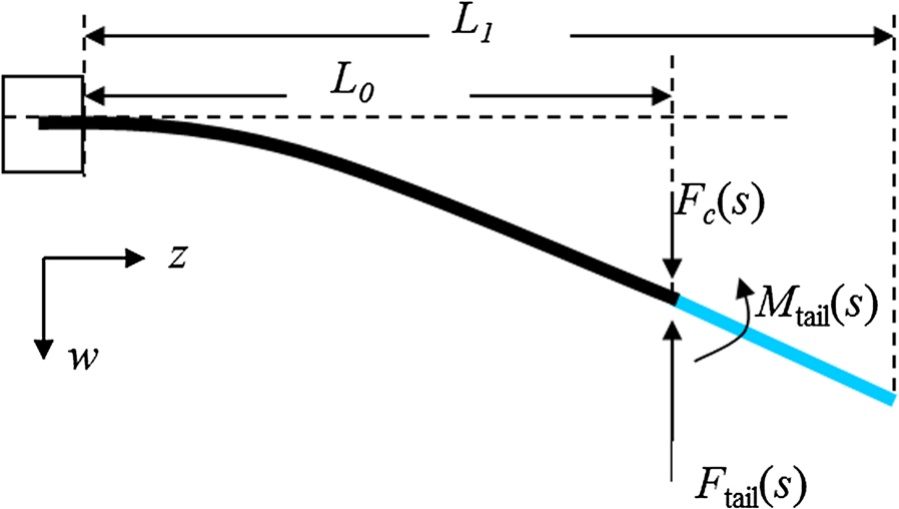
Forces and moments acting on the hybrid tail [33]

Figure 4 shows the forces and moments acting on the hybrid tail. The generalized force can be written as:

33 $$f_{2i} \left(s \right) = \frac{1}{M_i}\left({\int\limits_0^{L_0} {F_{\rm d} \left({z,s} \right)\varphi_i \left(z \right)\hbox{d}z} + \varphi_i \left({L_0} \right) F_{\rm tail} (s)} \right) + \frac{(M_{\rm tail}(s)+M(L_0,s))\varphi^{\prime}_i (L_0)}{M_i},$$

where *M*_tail_ an *F*_tail_ are defined in (19) and (20), respectively, *F*_d_(*z*, *s*) and *M*(*L*_0_, *s*) are defined in (31) and (32), respectively.

Then with the generalized force (33), one can solve the beam equation using the mode summation method (22). Finally, the transfer functions relating *w*(*L*_0_, *s*) to *V*(*s*) and that relating to $w^{{\prime }} (L_{0} ,s)(s)\mathop = \limits^{\Delta } \partial w(z,s)/\partial z|_{{z = L_{0} }}$ to *V*(*s*) can be found as

34 $$H_2 \left({L_0,s} \right) = \frac{{\left({1 + F_s} \right)A_s - B_sE_s}}{{\left({1 + C_s} \right)\left({1 + F_s} \right) - B_sJ_s}},$$

35 $$H_{2d} \left({L_0,s} \right) = \frac{{\left({1 + C_s} \right)E_s - A_sJ_s}} {{\left({1 + C_s} \right)\left({1 + F_s} \right) - B_sJ_s}},$$

where *A*_*s*_, *B*_*s*_, *C*_*s*_, *F*_*s*_, *J*_*s*_, and *E*_*s*_ are transfer functions related to the dimensions of the caudal fin. See [33] for the detailed derivation. From (18), (34), and (35), one can obtain the transfer functions relating the bending displacement and the slope at *z* = *L*_1_ to the voltage input *V*(*s*) as follows:

36 $$H_3 \left({L_1,s} \right)= \frac{{w\left({L_1,s}\right)}}{V\left(s \right)} = H_2 \left({L_0,s} \right) + H_{2d}\left({L_0,s} \right) D,$$

37 $$H_{3d} \left({L_1,s} \right) = \frac{{w^{\prime} \left({L_0,s} \right)}}{V\left(s \right)} = H_{2d}\left({L_0,s} \right).$$

### Speed model of robotic fish

Given a voltage input *V*(*t*) = *A*_m_sin(*ωt*) to the IPMC actuator, the bending displacement and the slope of the tail at the tip *z* = *L*_1_ can be written as

38 $$w(L,t)= A_m \left|H(j\omega)\right|\sin(\omega t +\angle H(j\omega)),$$

39 $$\frac{\partial w(z,t)}{\partial z}|_{z=L} = A_m \left|H_{d} (j\omega) \right| \sin(\omega t +\angle H_{d}(j\omega)),$$

where $\angle ( \cdot )$ denotes the phase angle, and *H*(*s*) and *H*_d_(*s*) represent *H*_3_(*L*_1_,s) and *H*_3d_ (*L*_1_, *s*), respectively. From (4), one can then obtain the steady-state speed *U* of the robotic fish under the square wave actuation voltage as

40 $$U = \sqrt {\frac{{m \cdot \frac{8\omega^2 A_m^2}{\pi^2}\sum\nolimits_{n = 1,3,5, \ldots}^\infty {\left| {H\left({jn\omega} \right)} \right|^2}}}{{C_D \rho_w S + m \cdot \frac{8A_m^2}{\pi^2}\sum\nolimits_{n = 1,3,5, \ldots}^\infty {\frac{{\left| {H_d \left({jn\omega} \right)} \right|^2}}{n^2}}}}}.$$

## Fabrication of IPMC actuating membrane capable of 3D deformation

3D kinematic motions have been observed from many types of biological fins, including pectoral fin and caudal fin [4]. To mimic the swimming behavior of fish, flapping only motion is not sufficient enough to generate high efficient propulsion and high maneuverability. Since IPMC can only generate bending motion, in this section, we present two different fabrication technologies that enable us to fabricate IPMC actuation membrane capable of generating 3D deformation. Comparison of these two approaches will be given based on the characterization results. Most of the work presented in this section was published in [41, 42].

### Lithography-based fabrication process

**Fig. 5:**
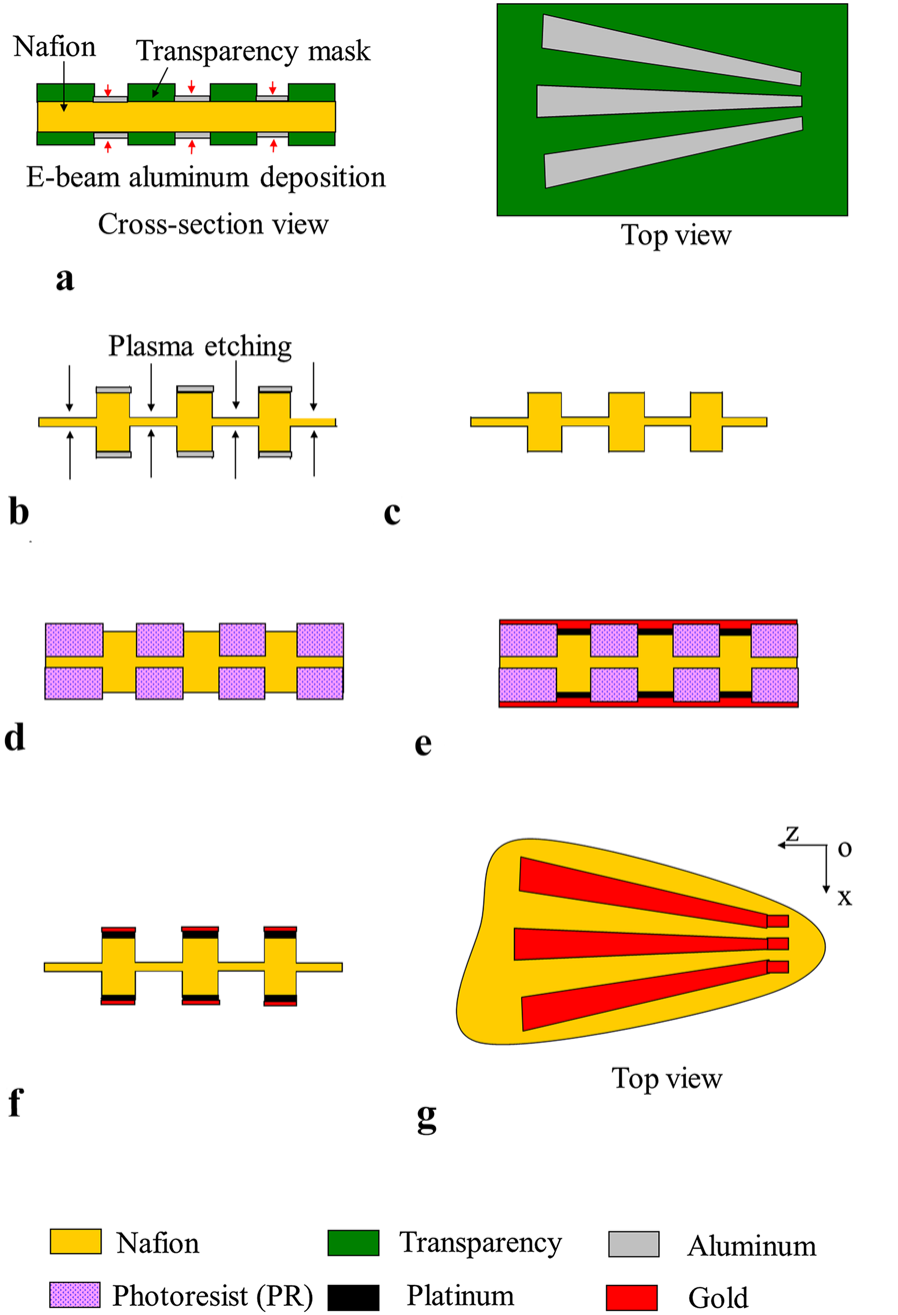
Lithography-based fabrication process [41]. **a** Deposit aluminum mask on both sides of Nafion film. **b** Thin down passive area with plasma etch. **c** Remove aluminum mask and perform ion-exchange to make Nafion stiffer. **d** Deposit PR and then pattern PR through lithography. **e** Perform another ion-exchange and electroless palting of platinum to create IPMC electrodes. **f** Remove PR and perform final treatment and **g** Cut the patterned IPMC into a fin shape

The first fabrication process is lithography-based, monolithic fabrication process for creating multiple IPMC regions that are mechanically coupled through compliant, passive membrane. Both the IPMCs and the passive regions are to be formed from a same Nafion film. There are two major challenges in fabricating such actuators. First, the passive areas can substantially constrain the motion of the active areas. An effective, precise approach is needed for tailoring the stiffness of the passive areas. Second, Nafion films are highly swellable in a solvent. Large volume change results in poor adhesion of photoresist to Nafion and creates problems in photolithography and other fabrication steps. To overcome these challenges, two novel fabrication techniques have been introduced: (1) selectively thinning down Nafion with plasma etch, to make the passive areas thin and compliant; (2) impregnating Nafion film with platinum ions, which significantly reduces the film swellability and allows subsequent lithography and other steps. Fabrication of an artificial pectoral fin is taken as an example. As illustrated in Fig. 5, the major process steps include:

1. Create an aluminum mask on Nafion with e-beam deposition, which covers the intended IPMC regions.
2. Etch Nafion with argon and oxygen plasmas to thin down the passive regions.
3. Remove the aluminum mask and place the sample in platinum salt solution to perform ion exchange. This will stiffen the sample and make the following steps feasible.
4. Pattern with photoresist (PR), where the targeted IPMC regions are exposed while the passive regions are protected.
5. Perform the second ion exchange and reduction to form platinum electrodes in active regions. To further improve the conductivity of the electrodes, 100-nm gold is sputtered on the sample surface.
6. Remove PR and lift off the gold on the passive areas. Soften the passive regions with HCl treatment (to undo the effect of step 3).
7. Cut the sample into a desired shape.

**Fig. 6:**
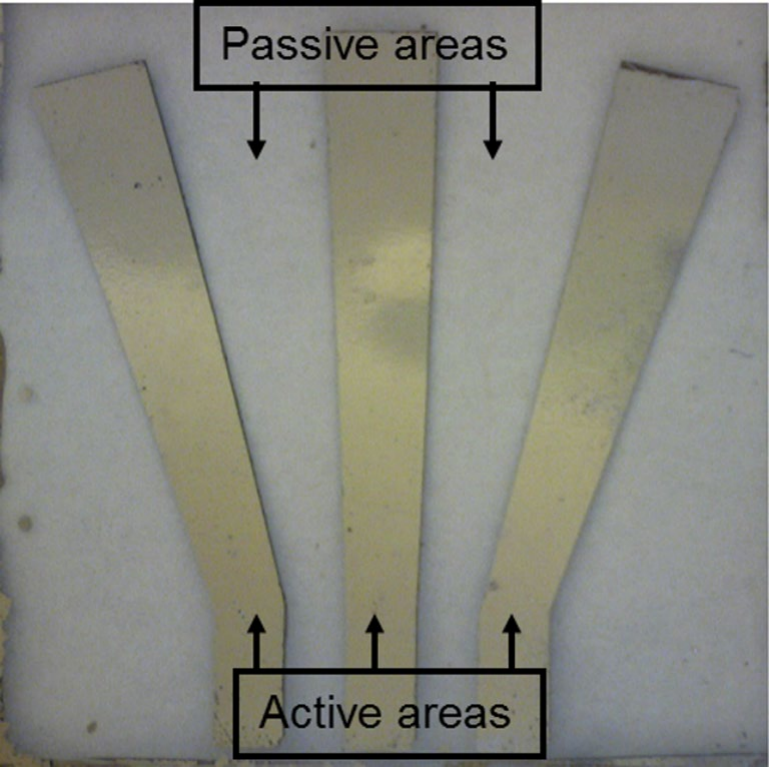
Fabricated pectoral fin based on lithograph-based fabrication process [41]

Based on the above process, a pectoral fin has been fabricated, which is shown in Fig. 6. The fin was able to generate complex deformations, including bending, twisting, and cupping, by controlling the phase angle among the signals applied to the active areas. The fin was characterized in terms of twisting angle and deflection. The fin was able to achieve 15 degree peak-to-peak twisting angle with about 2-mm bending displacement.

### Assembly-based PDMS bonding process

The second fabrication process is assembly-based PDMS molding fabrication process to create an IPMC-based actuating membrane, capable of complex 3D deformations. The first step in the process is to synthesize the IPMC actuator. Many groups have developed different IPMC fabrication processes to accommodate various functions [43–47]. In [42], Chen et al. followed most of the fabrication procedure outlined in [43] but add a multiple platinum plating process that reduces the surface resistance of the electrodes to improve the actuation performance [47].

**Fig. 7:**
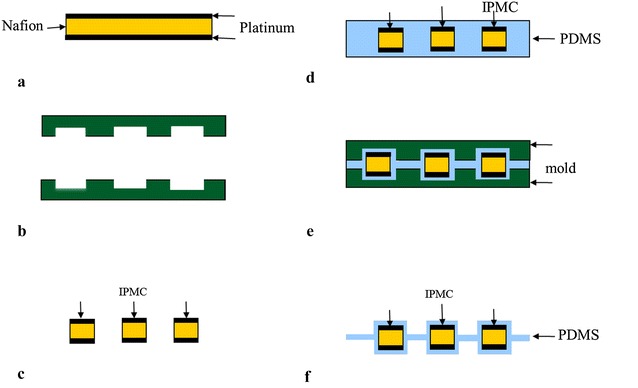
Assembly-based fabrication process [42].**a** Fabricate IPMC. **b** Make a mold. **c** Cut IPMC into seperated strips. **d** Cover the IPMCs with uncured PDMS gel. **e** Clamp the mold containinig the IPMCs and uncured PDMS, and then cure the PDMS at room temperature. **f** Remove the mold

**Fig. 8:**
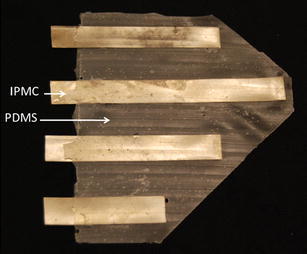
Fabricated IPMC artificial pectoral fin based on the assembly-based fabrication process [42]

The assembly process to produce an integrated IPMC/PDMS actuating membrane is shown in Fig. 7. A mold is fabricated from Delrin using a CNC rapid mill machine (MDX-650, Roland). The mold is designed to house the IPMC beams (280 μm thick), which is then surrounded with uncured PDMS gel (Ecoflex 0030, Smooth-on Inc.). The mold, containing the IPMCs and uncured PDMS, is clamped and the PDMS is allowed to cure at room temperature for 3 h. The mold is removed leaving the IPMC/PDMS membrane actuator (Fig. 8). The PDMS has a final thickness of 190 μm that is measured using a caliper (CD-S6”CT, Mitutoyo). The characterization of the actuating membrane has shown that the maximum twist angle can reach up to 15°, the flapping deflection can reach up to 25% of span-wise length, the tip force can reach up to 0.5 g force, and the power consumption is below 0.5 W.

## Bio-inspired design of underwater robots

Three types of bio-inspired underwater robots have been developed in this research, including robotic fish propelled by a caudal fin, robotic manta ray propelled by two pectoral fins, and robotic fish propelled by multiple IPMC fins. The robotic fish was fabricated to verify the speed model described in “Physics-based control-oriented modeling of robotic fish propelled by IPMC caudal fin” section while the manta ray was built to validate the fabrication process for pectoral fin described in “Bio-inspired design of underwater robots” section. The robotic fish propelled by multiple IPMC fins was developed to validate both forward swimming and turning capabilities. Most of the work presented in this section was published in [33, 42, 48, 49]

### Robotic fish propelled by caudal fin

**Fig. 9:**
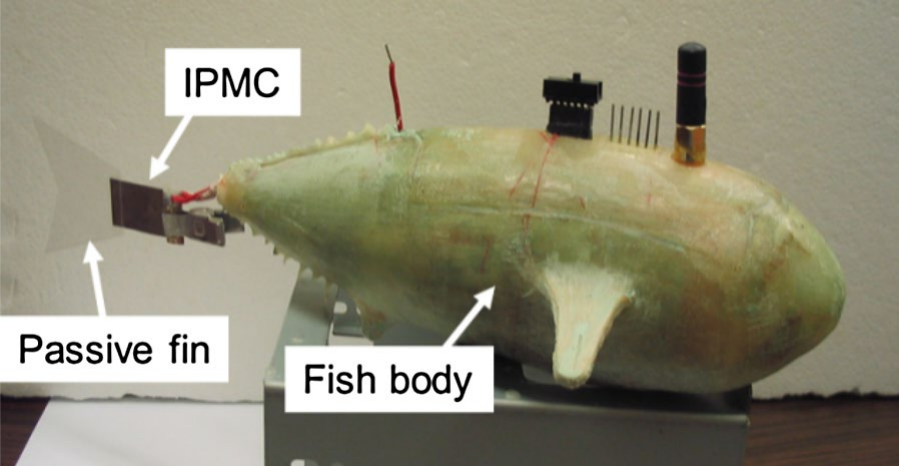
Robotic fish propelled by an IPMC caudal fin [33]

A robotic fish propelled by an IPMC caudal fin was developed in Tan’s group at Michigan State University, shown in Fig. 6 [33]. Inspired by biological fish fins, where passive, collagenous membranes are driven by muscle-controlled fin rays [7], a passive, plastic fin was attached to the tip of IPMC to enhance propulsion. It consists of a rigid body and an IPMC caudal fin (Fig. 9).

**Fig. 10:**
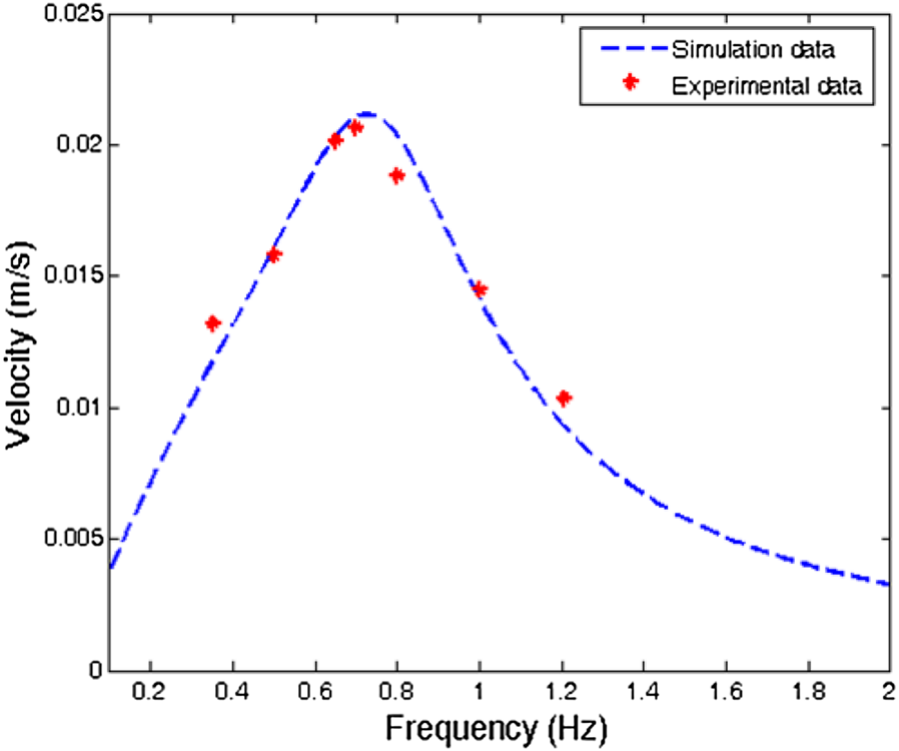
Speed versus flapping frequency [33]

The speed model has been validated through experiments. Four different types of caudal fins with different dimensions have been used in the robotic fish to verify the geometrical scalability of the speed model. A series of square wave signals with the frequency ranging from 0.2 to 2 Hz were applied to the tail to propel the fish. Figure 10 shows one set of data for the robotic fish with tail A. It shows that the experimental data can be captured by model prediction well. There was an optimal frequency at which the fish swam at its fast speed (0.02 m/s), which was 0.125 BL/s [33].

### Robotic manta propelled by pectoral fin

**Fig. 11:**
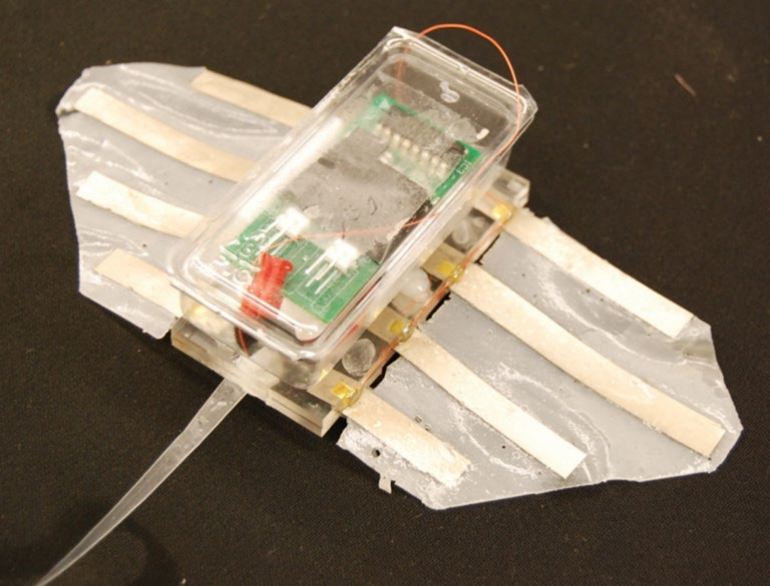
Free-swimming robotic manta ray [42]

Based on the assembly-based fabrication process, Chen et al. [42] developed a robotic manta ray using two pectoral fins. Two acrylic frames with gold electrodes were made to clamp the artificial wings to the body support. Gold electrodes were used to minimize corrosion. A polymer foam was put into the middle of the frame to make the robot slightly positively buoyant. The fully assembled robot is 8 cm long (not including the length of the tail), 18 cm wide, 2.5 cm high, and weights 55.3 g. The free-swimming robot is shown in Fig. 11. The total cost of the robot is about $200.

**Fig. 12:**
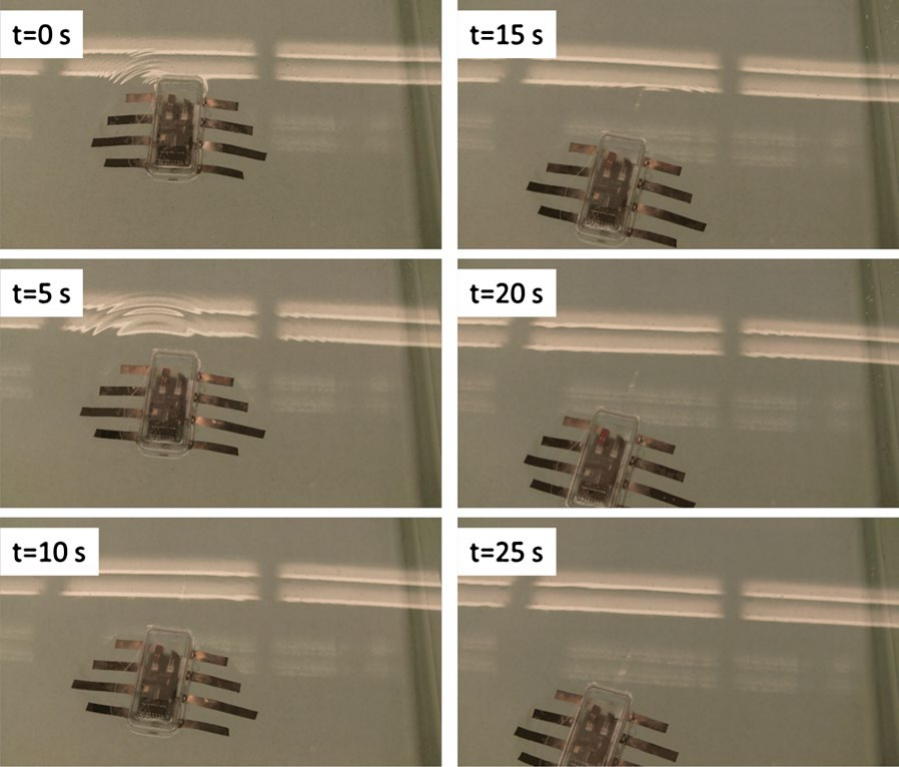
Snapshot of a swimming robotic manta ray [42]

The robot was tested in a water tank (1.5 m wide, 4.7 m long, and 0.9 m deep). As the first attempt, the operating frequency of the square wave actuation voltage is tuned at 0.4 Hz and the amplitude is set at 3.3 V. A digital video camera (VIXIA HG21, Canon) is used to capture the movies of the swimming robot. Figure 12 shows six snapshots of the swimming robot from top view. Each snapshot is taken every 5 s. One can extract the speed of the robot from the movie through the Edge Detection program in the Labview. The swimming speed shown in Fig. 12 is 0.42 cm/s. Since the body length is 8 cm, one can calculate the speed is 0.053 BL/s. This was believed to be the first demonstration of an IPMC-propelled free-swimming robotic batoid ray. It also validated the proposed fabrication process for making IPMC actuator capable of 3D kinematic motions.

**Fig. 13:**
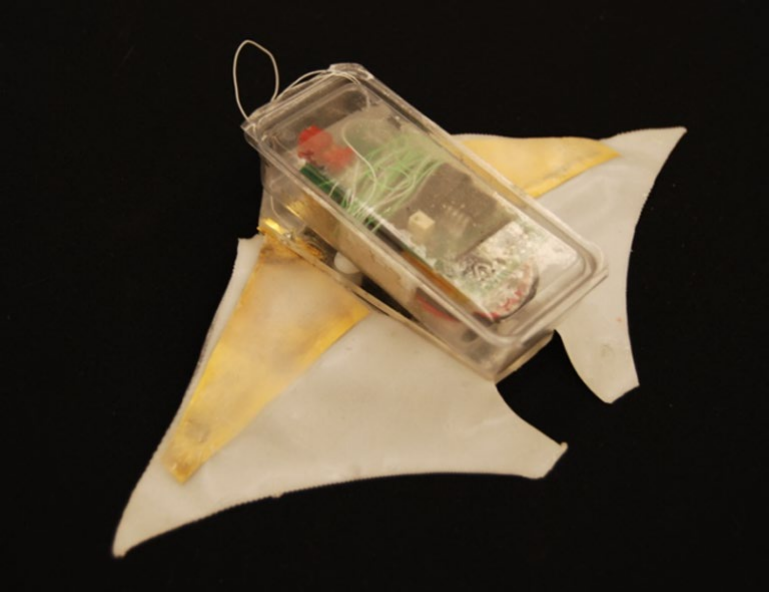
Free-swimming robotic manta ray with one IPMC placed at the leading edge of pectoral fins [48]

To simplify the control strategy, Chen et al. [48] developed a robotic manta ray with two pectoral fins where only one IPMC was placing at the leading edge. The fully assembled robot was 11 cm long, 21 cm wide, and 2.5 cm high with a mass of 55 grams. The free-swimming robot with the control unit is shown in Fig. 13.

**Fig. 14:**
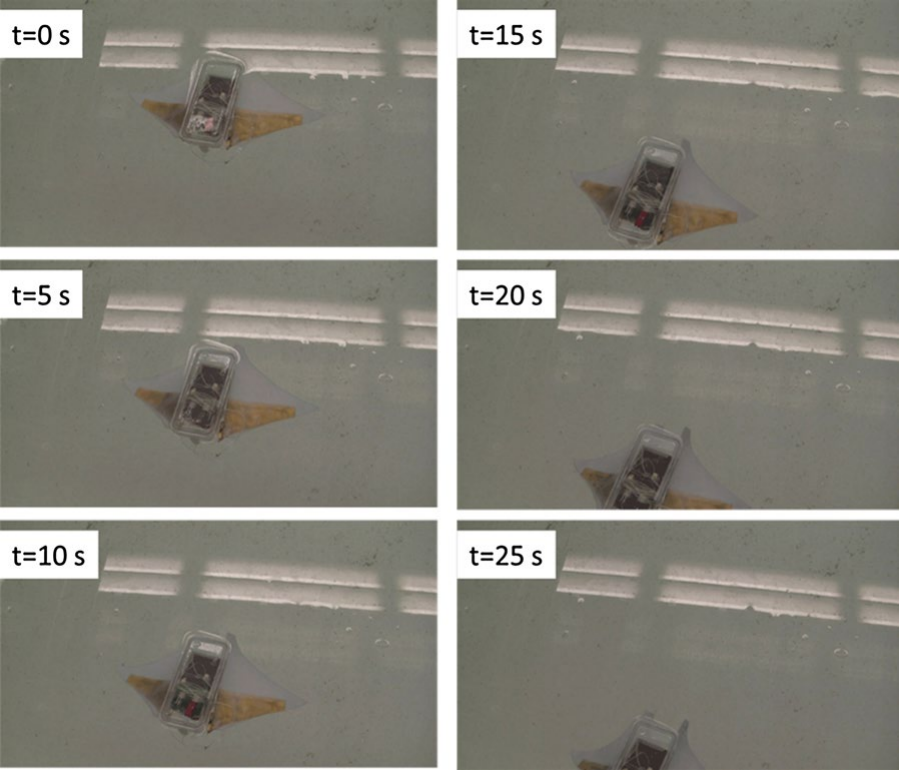
Snapshot of a swimming robotic manta ray [48]

Figure 14 presents six snapshots of the swimming robot from top view. Each snapshot was taken every 5 s. A swim speed of 0.74 cm/s was calculated from the movie using the Edge Detection program in the Labview. Since the body length was 11 cm, the speed in body length per second (BL/s) was 0.067.

### Robotic fish propelled by multiple IPMC fins

**Fig. 15:**
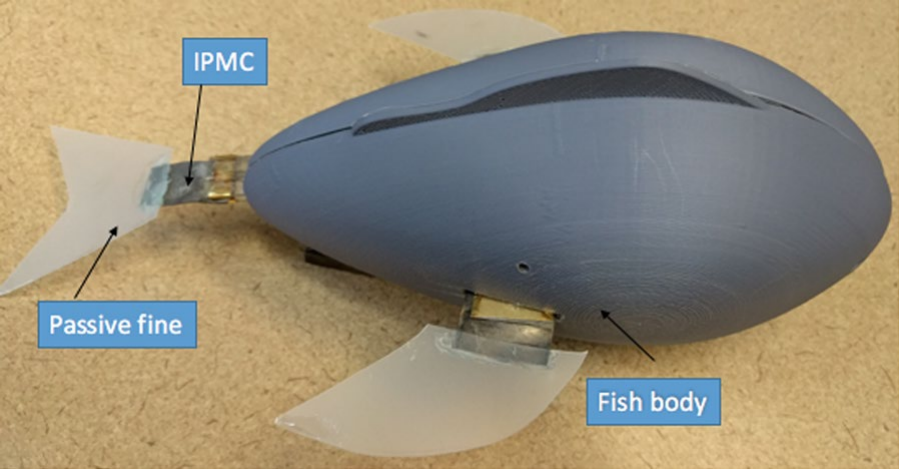
Assembled robotic fish propelled by multiple IPMC fins [49]

In the experiments of two robotic manta ray developed by Chen et al, it has been observed that if two pectoral fins were controlled differently, the turning performance of robotic manta ray is better than that of the robotic fish propelled by a caudal fin only. It would be good to utilize two pectoral fins for maneuvering and one caudal fin for main propulsion. Followed by this idea, Ye et al. [49] developed a robotic fish propelled by multiple IPMC fins. Figure 15 shows the assembled robotic fish, which was 18 cm long and 8 cm wide. The total weight of the robot was 290 g. Overall, the fish had slightly positive buoyancy.

**Fig. 16:**
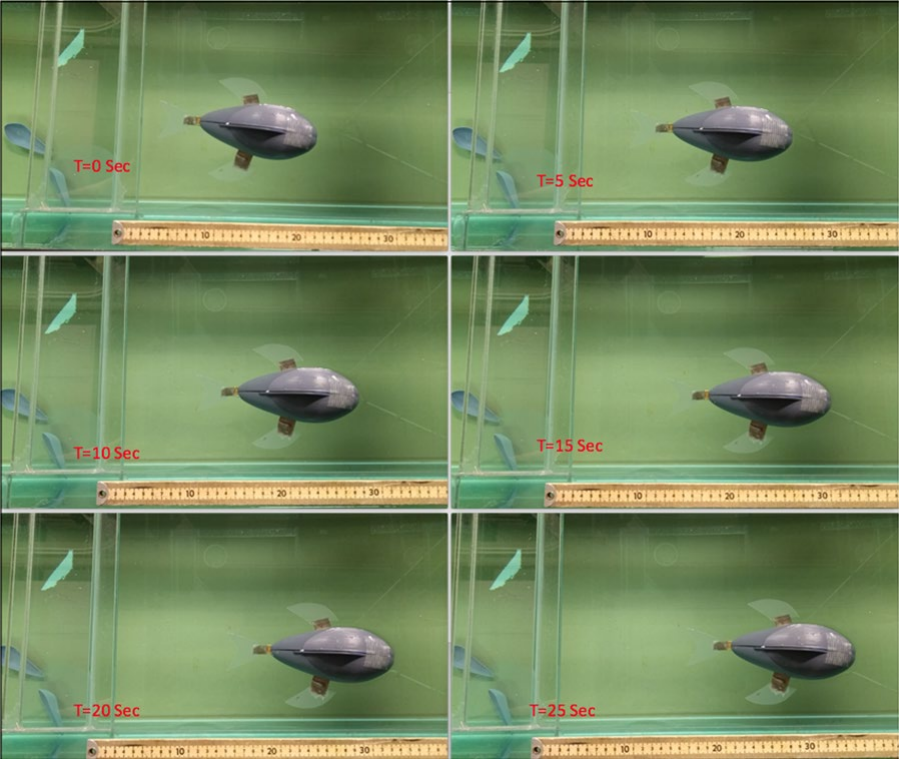
Snapshots of forward swimming test [49]

The fish’s forward swimming speed was controlled by changing the flapping frequency of the caudal fin. A square wave signal with 7.3 V magnitude and 0.55 Hz frequency was applied to the caudal fin. The pectoral fins were also actuated. The forward swimming speed reached about 0.067 BL/s. Also, there was a threshold whereby the frequency was neither too high nor too low for the fish to swim. Figure 16 shows the snapshots of a forward swimming test.

Turning tests were conducted to verify the steering capability of the pectoral fins. To make a left turn, the left pectoral fin was actuated with the same actuation signal applied to the caudal fin, while the right pectoral fin was kept inactive. The caudal fin provided the forward swimming direction, while the force generated by the left pectoral fin made the fish tail turn to the left.

**Fig. 17:**
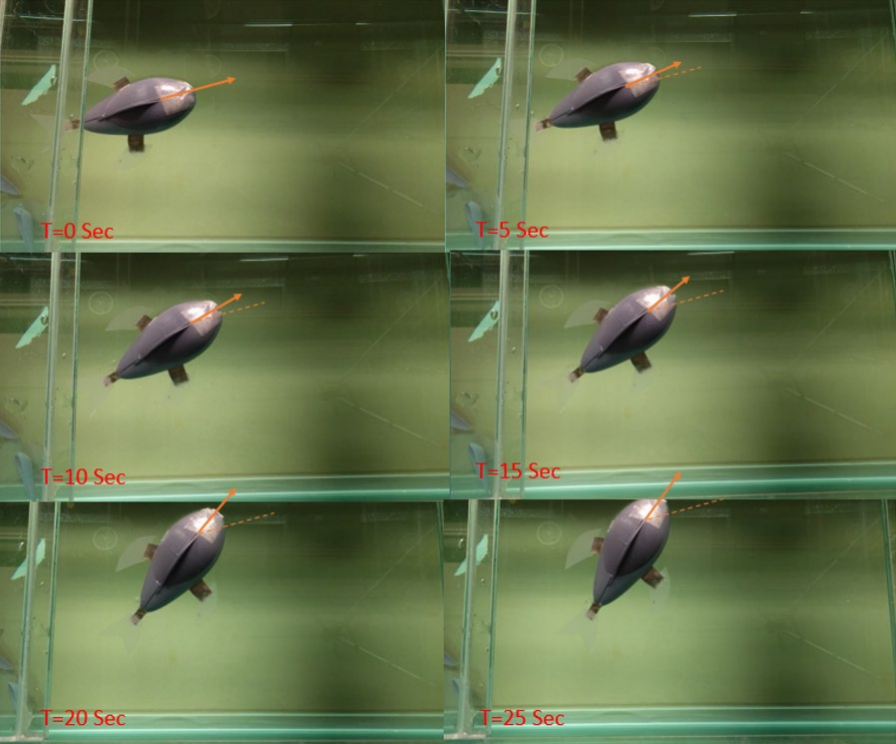
Snapshots of left-turning swimming test [49]

To make a right turn, the right pectoral fin was actuated with the same actuation signal applied to the caudal fin while the left pectoral fin was kept inactive. Actuation of the right pectoral fin made the fish turn to the right. Figure 17 shows the snapshots of a left-turning swimming test. The robot reached up to 2.5 degree/s turning speed.

## Discussion and conclusion

The physics-based and control-oriented model of the robotic fish will offer a great help in optimal design of the caudal fin and real-time control of the fish. It incorporates the hydrodynamics of the fish and actuation dynamics of IPMC. However, it can only capture the propulsion generated by bending only motion. If a 3D kinematic motion is generated by a caudal fin or pectoral fin, the model needs to be modified to capture the thrust created by the fin which will be also three dimensional. The thrust can be calculated by integrating the hydrodynamic force acting on the fin. It will be a great challenge to capture the fluid-to-soft-membrane interaction since the boundary conditions of the fluid dynamics PDE equation are more complicated since the shape of the membrane changes with time. An approximation method must be found to simplify the 3D modeling of complex pectoral fin or caudal fin, which would be a future direction in this research.

The fabrication process for creating an IPMC that is capable of 3D kinematic motion follows two different approaches. The lithography-based approach is able to create meso- or microsize pectoral fin and it is suitable for batch production. However, the passive area of the fin is still Nafion membrane, which is not as stretchable as PDMS material. The twisting angle of 3D kinematic motion is constrained by the passive area even the active areas are controlled differently. The assembly-based approach solves the problem in the passive areas since a soft and stretchable PDMS can be selected in those areas. However, the process is non-monolithic and unsuitable for batch production. The process is also unable to create meso- or microsize pectoral fin. As a conclusion, each process has unique advantages and disadvantages. Choosing which process for making pectoral fin depends on the size of the robot and its application. The future direction of fabricating 3D deformable membrane would be 3D printing other soft materials and Nafion film into a seamless and arbitrary-shaped membrane which will consist of active areas and passive areas. This printing process would be either scaled up or scaled down, which could print mesoscale or microscale fish fins. The challenges would be 3D printing of two different soft materials in one platform.

Three types of bio-inspired underwater robot were reviewed in this paper. The robotic fish propelled by a caudal fin shows a reasonable good swimming forward performance (0.125 BL/s), while the robotic manta ray shows a slow swimming forward performance (0.053 BL/s). The 2D maneuverable robotic fish propelled by multiple IPMC fins showed some maneuvering capabilities (forward speed 0.067 BL/s and 2.5 degree/s), which are not very promising. The possible reasons might be that the pectoral fin and caudal fin were not optimally designed and the body was not optimally designed. However, using IPMC only in robotic fish or robotic rays might not be a good idea since IPMC cannot generate high-frequency flapping which is really needed for high-speed swimming or quick turning. The future direction of bio-inspired robots design using IPMC would be combining both IPMC and other fast responsive actuators, such as electrical motors, to achieve both high speed and high maneuvering capabilities. The challenges would be bio-inspired design of a hybrid fish tail and dynamic modeling and control of the robot propelled by such hybrid tail.
